# MRI-guided Dose-escalated Salvage Radiotherapy for Bulky Bladder Neck Recurrence of Prostate Cancer

**DOI:** 10.7759/cureus.2360

**Published:** 2018-03-22

**Authors:** Amar U Kishan, Marguerite Tyran, Michael L. Steinberg, Stuart B Holden, Minsong Cao

**Affiliations:** 1 Department of Radiation Oncology, University of California, Los Angeles; 2 Department of Urology, University of California, Los Angeles

**Keywords:** salvage, prostate cancer, radiotherapy

## Abstract

Nearly 30% of patients treated with radical prostatectomy for prostate cancer ultimately develop biochemical recurrences, and nearly a quarter of men with nonpalpable biochemical recurrences have gross local recurrences identified with magnetic resonance imaging (MRI). The only curative intervention for patients with recurrent disease after radical prostatectomy is salvage radiotherapy – this is particularly true for patients with gross local recurrences. Furthermore, even in patients with an incurable metastatic disease, a local recurrence can be the source of significant morbidity and should be addressed.

Delivering a sufficient dose of radiation in the postoperative setting to control gross disease while minimizing toxicity poses a significant technical challenge. Because of the inherent uncertainty in the verification of gross disease positioning with standard onboard imaging technologies, large margins must be used. Larger margins, in turn, will lead to larger volumes of tissue receiving high doses of radiation, potentially increasing long-term toxicity.

Herein, we present the case of a patient with a bulky gross recurrence (>40 cm^3^) at the bladder neck and synchronous metastatic disease who was referred for salvage radiotherapy after a multidisciplinary consensus recommendation to pursue local therapy for mitigating urinary morbidity from the bulky tumor. The case illustrates the utilization of MRI-guided radiotherapy to allow significant margin reduction, thereby facilitating the delivery of an escalated dose of radiotherapy to a bulky recurrence.

## Introduction

Salvage radiotherapy (SRT) is the only known curative intervention for the 30% of men who experience a biochemical recurrence following radical prostatectomy (RP) for prostate cancer (PCa) [1]. Of patients referred for consideration of SRT based on rising prostate-specific antigen (PSA) alone, nearly 25% will have a gross recurrent disease that can be visualized with magnetic resonance imaging (MRI) [2]. In men with a grossly recurrent disease, SRT is important in offering local control regardless of whether the PCa has already metastasized or not. While common SRT dose/fractionation regimens range from 64.8-68 Gy in 1.8-2 Gy fractions, higher doses are thought to be associated with greater biochemical control [3]. Additionally, multiple randomized trials in the definitive setting have demonstrated a clear biochemical benefit to escalating doses in the order of 78 Gy in 2 Gy fractions [4], and it is reasonable to extrapolate that comparable doses would be required to control gross disease in the recurrent setting. As nearly 75% of post-RP local recurrences are located in the peri-anastomotic area or around the bladder neck, SRT dose escalation is limited by concerns of causing significant toxicity [5].

In this setting, MRI-guided radiation therapy presents several technical advantages, including superior soft tissue differentiation and compensation for interfraction and intrafraction movement with onboard imaging, gating, and/or adaptive planning [6]. Herein, we present the case of a patient with a bulky bladder neck recurrence (>40 cm^3^) threatening to affect urinary function. This patient was successfully treated with standard dose SRT to the prostatic fossa, followed by an MRI-guided focal boost to the bulky gross disease. Over 30 months later, he has radiographic evidence of significant regression and has maintained his pretreatment urinary function status with the addition of a detrusor muscle relaxant agent.

## Case presentation

**Figure 1:**
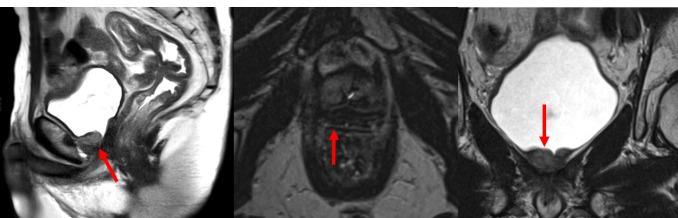
Diagnostic Magnetic Resonance Imaging of a Bulky Recurrence Diagnostic magnetic resonance imaging (MRI) depicting a bulky gross recurrence in the sagittal, axial, and coronal planes. All images shown are T2-weighted turbo spin echo sequence images obtained with a 3.0 Tesla scanner. The red arrows indicate the gross recurrence.

The patient underwent a retropubic radical prostatectomy at age 60 for high-risk prostate cancer (biopsy Gleason score 3+5=8, initial PSA 10.2 ng/mL, and cT3a disease). Final pathology showed a locally advanced node-positive disease, staged pT4N1, with pathologic Gleason score 3+5=8, positive surgical margins, and one lymph node positive out of seven removed. He began hormonal therapy with an anti-androgen and ultimately transitioned to a luteinizing hormone-releasing hormone agonist. Due to side effects, he began to take this intermittently and continued with this for nearly 14 years, at which point, his PSA began to rise at a rapid rate, increasing from 1.6 to 3.0 ng/mL over a short course of time. He had a negative 99-technetium bone scan and elected to undergo an ^11^C-acetate positron emission tomorograph (PET)/computed tomography (CT) scan, which showed mild avidity for ^11^C-acetate in the prostate bed, as well as a sclerotic pubic ramus lesion and a lumbar spine lesion. A dedicated MRI of the prostate showed an area of thickening in the bladder neck with intense enhancement and restricted diffusion, measuring 3 cm (Figure 1). It also confirmed the pubic ramus lesion.

On focused questioning, he did endorse a recent increase in nocturia as well as a slightly weaker stream and occasional stress incontinence; he had used one pad per day since the surgery. Despite the presence of metastatic disease and his advanced age of 74, it was determined at a multidisciplinary tumor board that the bulky nature of the local recurrence mandated local therapy in addition to the resumption of indefinite hormonal therapy with a luteinizing hormone-releasing hormone agonist.

There was significant concern that it would be difficult to safely deliver a sufficient dose of radiation (i.e., in the order of 78 Gy in 2 Gy fractions) to the bulky recurrent lesion without causing serious injury to the genitourinary diaphragm (GUD), despite the fact that 14 years had elapsed since his surgery. The delivery would be complicated by the fact that his bladder neck mass was poorly visualized on diagnostic quality CT imaging, implying that image-guided radiotherapy (IGRT) based on cone-beam CTs would be of even poorer quality. As such, despite the use of daily IGRT, it was presumed that the necessary planning target volume (PTV) margin to account for the interfractional motion and setup uncertainties would be large, further increasing the dose to critical adjacent structures, such as the GUD. Additionally, the patient was on anti-coagulant medication for a previous cerebrovascular event, and his cardiologist expressed trepidation toward the placement of fiducial markers or the performance of salvage brachytherapy, which might have been alternative approaches.

Thus, a provisional plan was made to plan for an initial course of 66 Gy to a standard postoperative prostatic fossa volume to be delivered by a linear accelerator (NovalisTX, BrainLAB AG, Feldkirchen, Germany). Prior to this, simulation scanning would be performed on both a standard CT scanner and a commercially available tri-^60^Co-MRI-guided RT platform (ViewRay, Inc., Cleveland, OH, USA). Provided the lesion was well-visualized on the ViewRay simulation, we would then obtain a second simulation after 50 Gy. If the tumor had regressed significantly, we would consider delivering a 12 Gy boost on either the NovalisTx or the ViewRay. If, as expected, the tumor had not regressed, we would deliver a 12 Gy boost with the ViewRay, harnessing the onboard MRI to utilize tighter PTV margins.

**Figure 2:**
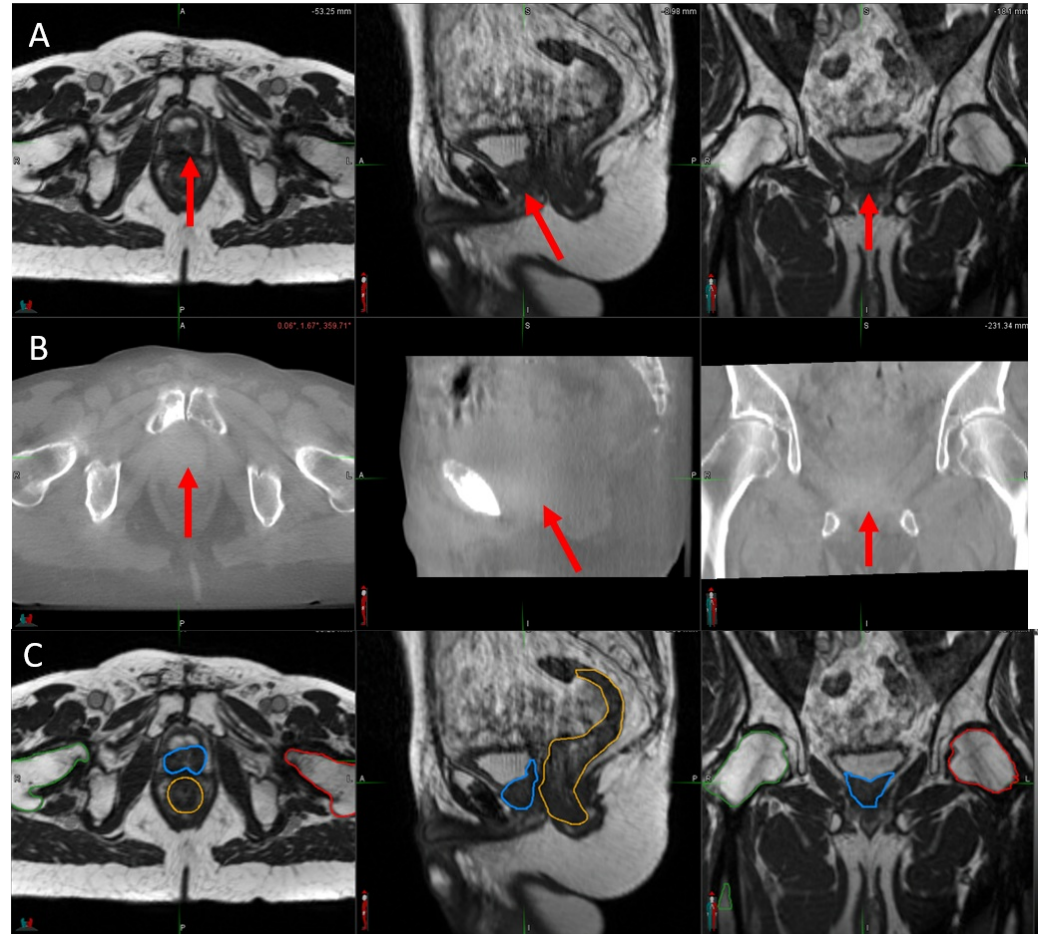
Image-guided Radiotherapy Images (A) Daily setup magnetic resonance imaging (MRI) scan obtained using the 0.35 Tesla ViewRay (ViewRay, Inc., Cleveland, OH, USA) system MRI, with a balanced steady state free precession (bSSFP) sequence. (B) Cone beam computed tomography imaging obtained using the onboard imager for the NovalisTx (BrainLAB AG, Feldkirchen, Germany) linear accelerator. In both panels (A) and (B), the red arrows indicate the location of the bulky recurrence. (C) Simulation bSSFP sequence MRI obtained on the ViewRay system, with the gross recurrent lesion depicted in blue, the rectum in orange, and the right and left femoral heads in green and red, respectively.

Simulation and daily setup MRI scans were obtained using the same imaging protocol on the 0.35 Tesla ViewRay MRI. A balanced steady state free precession (bSSFP) sequence image was obtained to acquire the 3D MRI images with 1.5 mm isotropic spatial resolution and a field of view (FOV) of 50x45x43 cm without contrast injection. The total acquisition time was 172 seconds. At the time of SRT simulation, the lesion was easily visible on the ViewRay MRI system and poorly visible on daily cone beam CT imaging for the linear accelerator (LINAC)-based treatment (Figure 2).

**Figure 3:**
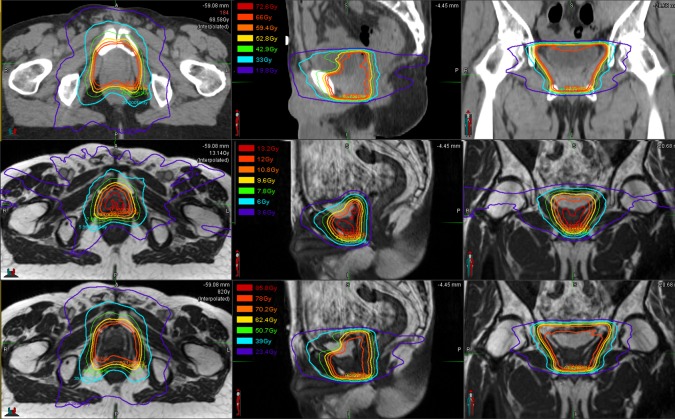
Initial, Boost, and Sum Plans The top row presents isodose curves for the 66 Gy initial prostatic fossa salvage radiotherapy plan, delivered with a NovalisTX (BrainLAB AG, Feldkirchen, Germany) linear accelerator. The middle row presents the isodose curves of the 12 Gy boost plan, delivered with the ViewRay (ViewRay, Inc., Cleveland, OH, USA) system. The bottom presents the sum total isodose curves overlaid on the ViewRay system simulation scan. The isodose line legend is featured in the middle panel of each row.

Therefore, after 25 fractions, a ViewRay simulation was obtained, confirming no significant regression of the lesion. At this time, the GTV measured 4.0x2.7x4.0 cm^3^. A 12 Gy plan was developed. Isodose curves for the original 66 Gy plan, 12 Gy boost, and summation are shown in Figure 3. The patient successfully completed the 12 Gy boost. He reported only a transient increase in urinary frequency.

**Figure 4:**
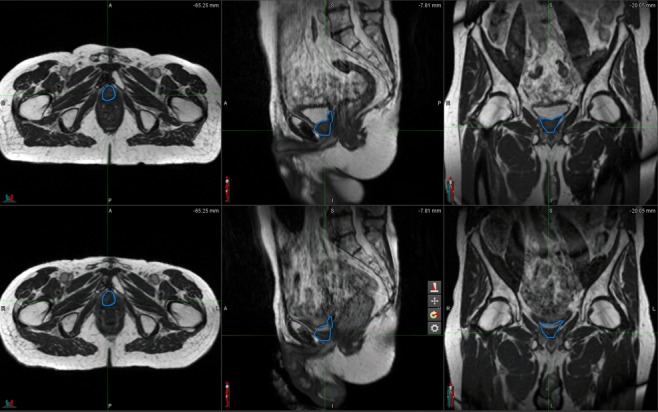
Initial and 30-month Follow-up ViewRay Magnetic Resonance Imaging of the Recurrent Disease The top row presents the initial ViewRay (ViewRay, Inc., Cleveland, OH, USA) system magnetic resonance imaging (MRI) in three planes, with the gross recurrence outlined in blue. The second row presents a 30-month follow-up ViewRay system MRI in three plan, with the same blue contours from the initial plan overlaid. This demonstrates a significant radiological response.

At one year following SRT, he did begin to develop more frequent urination, particularly at night, and was placed on mirabegron with a resolution of his symptoms. At his 30-month followup, imaging was obtained on the ViewRay device and indicated a significant regression of the bulky recurrent tumor (Figure 4). Despite an early PSA response, he did develop a progressive distant disease six months after SRT. Enzalutamide was initiated and his PSA has responded appropriately and remains at low levels.

## Discussion

This case presents a situation in which the improved soft tissue resolution afforded by the onboard MRI IGRT was leveraged to deliver an escalated radiotherapy dose to a bulky local recurrence, resulting in excellent local control and the prevention of severe obstructive urinary symptoms. While the patient did develop increased urinary frequency, requiring oral medication, he had impaired urinary function at baseline from his surgery and more severe urinary symptoms either from tumor progression or from radiation-induced toxicity were avoided. While prior reports of MRI-guided dose-escalated SRT exist, these previous studies used diagnostic MRIs to guide target delineation but did not utilize an MRI-guided RT unit. Additionally, many of the treated lesions were likely to be smaller than the bulky recurrences treated in this case. For example, investigators from the Hôpitaux Universitaires de Genève reported favorable outcomes for 131 patients who were treated with 64-64.4 Gy to a postoperative clinical target volume, followed by a boost to the gross residual disease (defined based on an endorectal coil MRI) to 72-78 Gy [7]. Eighty of these patients (61%) were treated with an a priori idealized target volume measuring approximately 2.4x2.4x3.6 cm^3^, which is <50% of the volume of the GTV in the present report (4.0x2.7x4.0 cm^3^). Hence, while the distribution of GTV dimensions in that experience was not reported, it is reasonable to presume that the boost GTV for the majority of patients was not larger than the clinical target volume (CTV) that received a nonescalated SRT dose and, therefore, the lesion treated in the current case presentation is significantly bulkier than those in the Geneva experience. A second case series of two patients treated with a focal boost to 76 Gy for gross disease identified on MRI similarly describes much smaller lesions [8].

While toxicity rates overall were favorable, the authors did note that receiving a boost was independently correlated with grade ≥2 late genitourinary toxicity by the Common Terminology Criteria for Adverse Events (CTCAE) v. 3.0 scale on a multivariate analysis. In the current report, the patient did experience grade 2 dysuria by the same toxicity metric but did not develop any grade 3 toxicity.

It is also notable that the GTV to PTV margin used in the Geneva experience was 15-20 mm, as opposed to the 5-mm margin used in the present report. This is likely multifactorial. First, MRI-based IGRT provides significantly superior soft tissue contrast than what conventional, onboard IGRT can provide, as shown in Figure 2, which, in turn, allows a reduction in PTV margins. Second, it is known that an endorectal coil can introduce anatomic distortions, and if the coil is not present at the time of radiotherapy, there is inherent setup uncertainty with regards to the location of the residual disease requiring a boost. It is likely that the favorable toxicity outcome in the present case is related to the narrow PTV margins that were utilized as a result of true MRI-guided radiotherapy. The validity of the 5-mm margin was confirmed, as tracking was performed during the treatment to assess for intrafractional motion. The intrafractional motion was confirmed to be less than 2 mm; however, due to the uncertainties inherent to setup, as well as the onboard imaging quality, it was thought that a 2-mm margin would be too narrow.

With regards to oncologic efficacy, SRT was pursued in this case purely to offer local control – as the patient had a synchronous metastatic disease, it would, by definition, not be curative. However, controlling gross disease in patients without metastatic disease would be pursued for curative intent. A long-term update of the Geneva experience reported a 5-year, biochemical, recurrence-free survival rate of 68.4% among patients treated with a boost, despite a mean PSA of 2.0 ng/mL, which compares favorably with historical control rates [9]. Because SRT is the only curative intervention in this patient population, and because the doses of SRT required to control a gross disease are presumably higher than the doses typically used to treat a microscopic disease, delivering dose-escalated SRT for a gross disease is critical for achieving a desirable oncologic outcome.

## Conclusions

SRT is the only known curative intervention for patients with recurrent prostate cancer after RP, and it is critical for achieving local control in patients with bulky recurrences. Safely delivering the requisite dose of radiation is complicated by concerns regarding toxicity and challenges with target localization. The superior soft tissue resolution of MRI-guided radiotherapy allows a significant reduction in PTV margin, thereby allowing safe and effective SRT dose escalation. Following the successful treatment of the patient presented in this report, we have treated several more patients with dose-escalated SRT to a gross disease and are developing a clinical protocol for this.
